# Near infrared/ red light therapy a potential countermeasure for mitochondrial dysfunction in spaceflight associated neuro-ocular syndrome (SANS)

**DOI:** 10.1038/s41433-024-03091-4

**Published:** 2024-05-03

**Authors:** Ethan Waisberg, Joshua Ong, Mouayad Masalkhi, Andrew G. Lee

**Affiliations:** 1 Department of Ophthalmology, University of Cambridge, Cambridge, UK; 2 Department of Ophthalmology and Visual Sciences, University of Michigan Kellogg Eye Center, Ann Arbor, MI USA; 3 School of Medicine, University College Dublin, Dublin, Ireland; 4 Department of Ophthalmology, Blanton Eye Institute, Houston Methodist Hospital, Houston, TX USA; 5 The Houston Methodist Research Institute, Houston Methodist Hospital, Houston, TX USA; 6 Departments of Ophthalmology, Neurology, and Neurosurgery, Weill Cornell Medicine, New York, NY USA; 7 Department of Ophthalmology, University of Texas Medical Branch, Galveston, TX USA; 8 University of Texas MD Anderson Cancer Center, Houston, TX USA; 9 Texas A&M College of Medicine, Houston, TX USA; 10 Department of Ophthalmology, The University of Iowa Hospitals and Clinics, Iowa City, IA USA

**Keywords:** Medical research, Diseases

## Introduction

Spaceflight-associated neuro-ocular syndrome (SANS) is a syndrome know to affect a significant proportion of astronauts during long-duration spaceflight (LDSF). SANS is characterized by optic disc edema [1], hyperopic shifts [2], chorioretinal folds [3] and globe flattening [4]. While the precise etiology of SANS remains incompletely understood, it is hypothesized to be as a result of the unique conditions encountered in the microgravity environment characteristic of LDSF, and heightened radiation exposure [5]. So far, SANS has been associated with alterations in fluid distribution and ocular structural adaptations induced by microgravity conditions [5].

The effects of microgravity and heightened exposure to radiation through galactic cosmic radiation and solar particle events during space missions are recognized in part for their notable influences on mitochondrial function [6]. Recent comprehensive analyses utilizing multi-omics revealed a consistent manifestation of mitochondrial stress during spaceflight [7]. These alterations include disturbances in metabolic pathways and gene regulation, and suggest that mitochondrial dysfunction plays an important role in prolonged space missions and SANS [7]. Oxidative stress emerges from an imbalance between reactive oxygen species (ROS) production and cellular antioxidant capacity [8]. Mitochondria act as both generators and receivers of ROS, and thus any impairment in their function may potentially contribute to the oxidative stress evident in SANS [8].

Retinal photoreceptors have an abundance of mitochondria to enable phagocytosis and outer segment renewal [9]. Retinal ganglion cell axons also have highly concentrations of mitochondria to efficiently transmit visual information from the eye to the brain. Unlike most cell types, neurons have absolute levels of mitochondrial function requirements to survive due to membrane potential generation requirements [10]. Further understanding the possible role of mitochondrial dysfunction in SANS is essential to develop countermeasures to mitigate the ophthalmic risks associated with long-duration spaceflight [11]. Current proposed countermeasures for SANS include intraocular pressure increase with swimming goggles [12], and vision enhancement with augmented reality to restore any visual losses that may occur [13, 14], however these current countermeasures are insufficient at addressing the underlying pathophysiology of SANS.

**Fig. 1:**
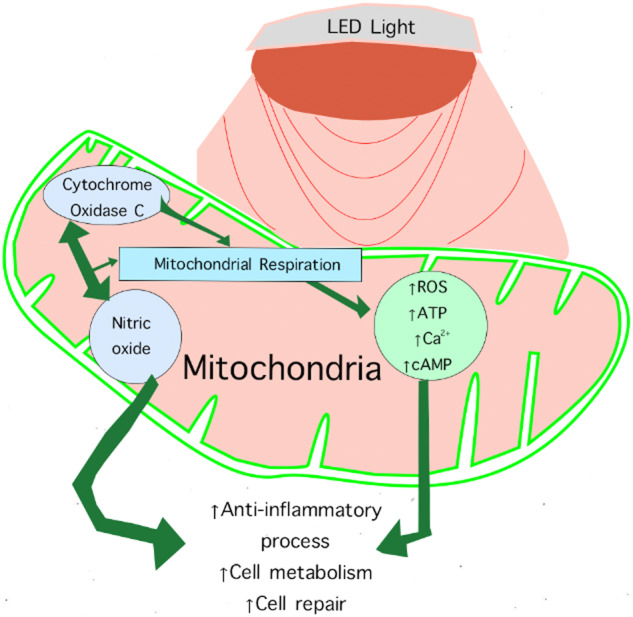
Hypothesized beneficial effects NIR/red light on mitochondrial function. These beneficial effects may potentially be useful to mitigate the symptoms of spaceflight associated neuro-ocular syndrome.

Red/Near Infrared (NIR) Light Therapy may serve as a non-invasive and side-effect-free countermeasure to improve mitochondrial function during LDSF. NIR/red light therapy is a form of phototherapy that utilizes specific wavelengths of light to promote healing and reduce inflammation [15, 16]. NIR/red light therapy is believed to enhance mitochondrial function via the absorption of photons by mitochondrial chromophores, such as cytochrome c oxidase, contributing to improved cellular respiration and ATP production, and reducing ROS generation [17]. NIR/red light also has known anti-inflammatory properties, which could mitigate inflammation associated with SANS and its impact on ocular tissues [17]. Furthermore, secondary effects can also occur hours following NIR/red light exposure including the dissociation of nitric oxide from its binding site, modification of the ATP pool and an increased mitochondrial intermembrane potential (Fig. 1) [18]. Alterations in ATP levels can affect cyclic adenosine monophosphate levels, intracellular calcium levels and cellular metabolism.

NIR/red light therapy has previously been shown to have potential in treating a wide variety of ophthalmic diseases that have a mitochondrial dysfunction component, such as: age related macular degeneration [19], diabetic macular edema [20], myopia [21], dry eye disease [22] and retinitis pigmentosa [23]. NIR/red light therapy has also been shown to improve the normal decline in mitochondrial function that is associated with aging, with a recent study finding improved contrast sensitivity for the tritan axis in participants aged 40-years-old and above treated with 670 nm light [24]. Another study conducted on healthy participants aged 55 years-and-older found that treatment with 670 nm light led to significant improvements in scotopic thresholds, but did not significantly alter visual acuity, low luminance visual acuity, rod-intercept time and no structural alterations on OCT [25].

While promising, the efficacy of red/NIR light therapy for SANS requires further investigation. Many of the existing studies involve smaller sample sizes and several of these studies lack control groups. Factors such as optimal light parameters, treatment duration, and long-term effects need to be carefully evaluated in order to develop standardized protocols for space missions. Moreover, integrating red/NIR light therapy into the comprehensive management of SANS alongside other interventions may offer a synergistic approach to mitigating ocular complications in astronauts. Finally, further research is required to fully understand the subtle functional and structural changes that occur in the eye during LDSF, to fully understand how to mitigate these effects [26, 27].
